# Two Atypical Cases of Hantavirus Infections from Sri Lanka

**DOI:** 10.1155/2018/4069862

**Published:** 2018-04-19

**Authors:** N. D. B. Ehelepola, B. M. L. S. Basnayake, S. M. B. Y. Sathkumara, K. L. R. Kaluphana

**Affiliations:** The Teaching (General) Hospital–Kandy, Kandy, Sri Lanka

## Abstract

There are two categories of hantaviruses resulting in two distinct illnesses. The Old World (Asia and Europe) viruses give rise to hemorrhagic fever with renal syndrome (HFRS), and the New World (Americas) viruses cause hantavirus pulmonary syndrome (HPS). Hantavirus infections have very similar clinical pictures and epidemiology to leptospirosis. Here, we present two cases of hantavirus infections from Sri Lanka (in South Asia) initially misdiagnosed as leptospirosis and later further investigated and diagnosed as hantavirus infections with serological confirmation of the diagnosis. They had clinical pictures of a combination of both HFRS and HPS as well as the involvement of the central nervous system. Hantavirus infections are rarely diagnosed in South Asia. Reports on such atypical clinical pictures of hantavirus infections are extremely rare. Having arrived at the correct diagnosis late/retrospectively, both these patients recovered notwithstanding being seriously ill, indicating adequate supportive therapy can save lives in such cases. The emergence of the hantavirus, an infection seriously affecting multiple organ systems with a high case fatality rate that is spread by aerosol and other routes, could become a serious public health issue in Sri Lanka.

## 1. Introduction

Hantaviruses are emerging zoonotic infections. People are incidental hosts. More than 40 hantavirus species are currently known and 22 species belonging to two categories cause two distinct illnesses in people [1–3]. One of those two is Old World (Asia and Europe) viruses that give rise to hemorrhagic fever with renal syndrome (HFRS), and the other is the more recently found New World (Americas) viruses causing hantavirus pulmonary syndrome (HPS), also known as hantavirus cardiopulmonary syndrome (HCPS) [1–3]. Hantavirus is a robovirus [1, 2, 4]. The hantavirus type corresponds with disease severity [1]. Rodents, soricomorphs, and bats have been known to host hantaviruses [2, 3]. Infected mammals shed viruses in their urine, feces, and saliva, and people can become infected via the aerosol route or via a breached skin or a mucus membrane [1–3]. Hantavirus infections have very similar clinical pictures and epidemiology to leptospirosis, another more common emerging bacterial zoonosis [5]. Medical laboratories of hospitals in Sri Lanka and in most other developing nations do not have the facilities to serologically diagnose both hantavirus infections and leptospirosis [5]. Therefore, hantavirus infections tend to be underdiagnosed and misdiagnosed as leptospirosis.

Here, we present two cases of hantavirus infection initially misdiagnosed as leptospirosis and later further investigated and diagnosed as hantavirus infections. They had clinical pictures of a combination of both HFRS and HPS and the central nervous system (CNS) involvement as well. Both cases were serologically confirmed as hantavirus infections.

## 2. Case Presentation

We illustrate the temporal sequence of the salient points of the illness of each case by a timeline followed by a detailed description.

### 2.1. Case 1

**Figure 1:**
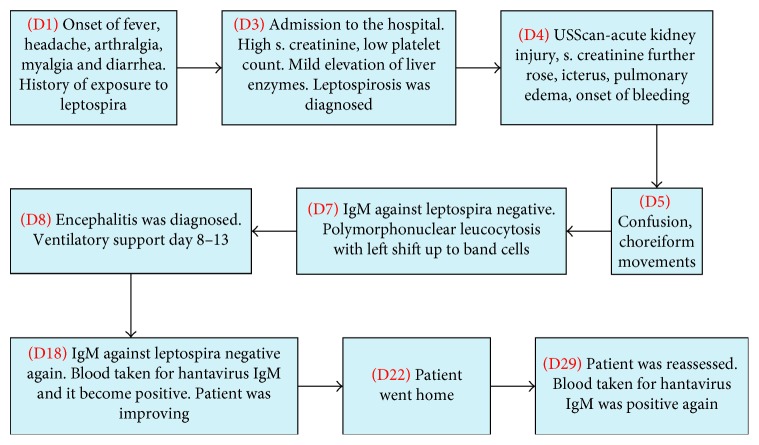
The timeline of salient events of case 1.

Figure 1 depicts the timeline of salient events of case 1.

**Figure 2:**
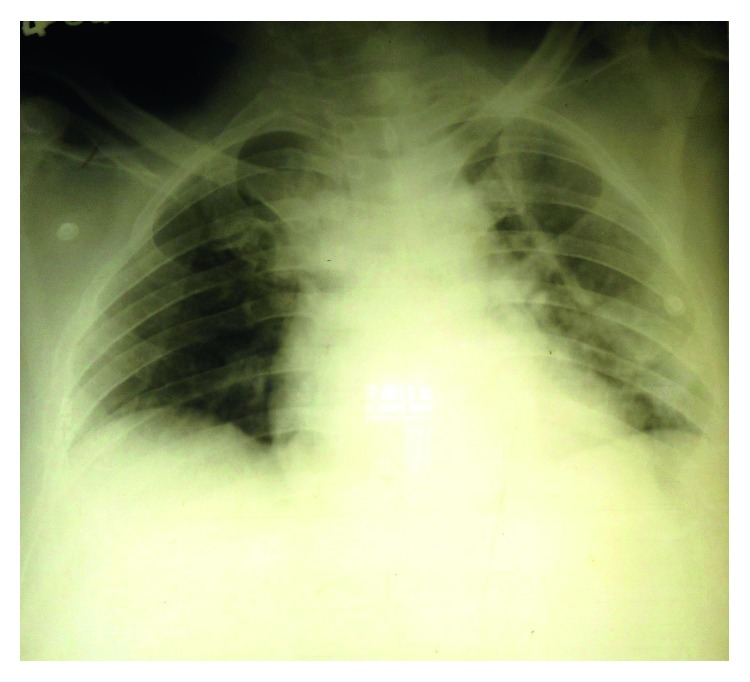
Chest X-ray of the patient of case 1 taken on day 6 of the illness depicting bilateral patchy opacities more in the peripheries in all three zones of both lungs indicating pulmonary edema due to a noncardiac cause. There is a bilateral small pleural effusion as well.

A 46-year-old previously healthy Sinhalese man from the district of Kandy was presented to us on the third day (day 3) of the illness with a fever, headache, arthralgia, and myalgia. On days 1 and 2 of the illness, he had watery diarrhea eight times and vomited twice. We used to get many patients with such symptoms during the time of his presentation; most of them were later diagnosed as dengue. Dengue and leptospirosis are prevalent in his locality, and he was used to working barefoot at a rice paddy, the usual local risk factor for exposure to leptospirosis. Physical examination revealed a body temperature of 38.9 degrees Celsius, conjunctival suffusion, a pulse rate of 100/minute, and blood pressure 100/60 mmHg, and cardiovascular, respiratory, and abdominal examination findings were unremarkable. Blood and urine were obtained for a workup. The NS1 antigen test for dengue was negative. His leukocyte count was 10.57 × 10^9^/l with 89.4% neutrophils, platelet count was 99 × 10^9^/l, serum creatinine was 1.3 mg/dl, serum sodium was 135 mmol/l, and potassium was 3.4 mmol/l. ALT was 56 U/l. Our provisional diagnosis was leptospirosis, and we initiated supportive therapy and intravenous (IV) ceftriaxone. IV furosemide and omeprazole was also started considering the rising creatinine level and to prevent stress ulcers in the stomach. On day 4, he again had watery diarrhea and developed mild dehydration. Despite being resuscitated with intravenous normal saline, his urine output reduced. He developed progressive dyspnea and bilateral lung crepitations. His blood pressure reduced to a nadir of 80/50 mmHg for a brief period. IV dobutamine was infused to maintain his blood pressure. Serum creatinine rose to 3.54 mg/dl, an ultrasound scan (USS) of the abdomen revealed bilateral renal parenchymal disease, and a repeated scan 4 days later demonstrated acute liver parenchymal disease as well. Leptospirosis with acute kidney injury (Weil's disease) was diagnosed and managed accordingly. His ECG (EKG) and troponin I level were normal. On day 5, his leukocyte count was 17.8 × 10^9^/l. His serum creatinine had risen to a peak of 4.48 mg/dl on the same day and then gradually decreased. The patient had fever spikes until day 15. He developed confusion and disorientation and choreiform movements of both hands on day 5. To cover possible viral meningoencephalitis, IV acyclovir (aciclovir) was started. We considered the possibility of hepatic encephalopathy, and IV metronidazole and thiamin were also added and lactulose syrup was prescribed. Methylprednisolone 1 mg was administered intravenously daily from day 5 to 7. His Glasgow coma scale (GCS) was between 12 and 14 from day 5 to 15. He developed icterus on day 4, and his serum bilirubin rose to a peak of 103.8 *μ*mol/l on day 6 and then declined. His ALT level rose to a peak of 134.3 and AST level rose to 75.2 in day 9 and then came down. His platelet counts were lowest (41–38 × 10^9^/l) on days 4 and 5 and then rose. He had gum bleeding and small subconjunctival hemorrhages. Considering his reducing GCS, abnormal behavior (excess smiling and restlessness) and choreiform movements, an NCCT brain was done on day 6 and that was normal. Cerebrospinal fluid (CSF) showed protein 260 mg/dl, 20 leucocytes (90% of which were lymphocytes), and 2800 red cells per mm^3^ on day 10. An EEG was done on day 8, and a consultant neurologist had assessed him with that and said the findings were compatible with encephalitis. His chest X-ray depicted evidence of pulmonary edema (Figure 2).

The patient's dyspnea aggravated and we could not maintain oxygen saturation with oxygen via facial mask. Hence, he was intubated and was on ventilatory support from day 8–13 at an intensive care unit (ICU). A 2D echocardiogram done on day 15 was normal.

An ELISA test done for IgM and IgG against leptospira on day 7 and repeated on day 18 delivered negative results. At the ICU, his leucocyte count increased to a peak of 33.4 × 10^9^/l on day 10 with 81.1% neutrophils. His day 4 CRP level of 256.4 mg/l dropped to 20.8 mg/l on day 7. A blood picture done on the same day showed polymorphonuclear leucocytosis with a left shift up to band cells. Even though repeated blood cultures (days 5, 9), urine culture (day 8), and endotracheal tube aspirate cultures (days 10, 11) were negative, ceftriaxone and metronidazole were continued for 14 days. The patient gradually recovered with supportive therapy and was discharged on day 22 of the illness. The ELISA test for IgM against hantavirus was positive on blood samples taken on day 18 (1 : 100 dilution) and day 29 (1 : 100 and 1 : 500 dilutions). A part of the day 18 sample was sent to Hokkaido University, Japan, for confirmation and that also was positive for IgM against the hantavirus. However, the indirect immunofluorescent antibody test (IFA) for IgG against the hantavirus was negative on the day 29 sample.

### 2.2. Case 2

**Figure 3:**
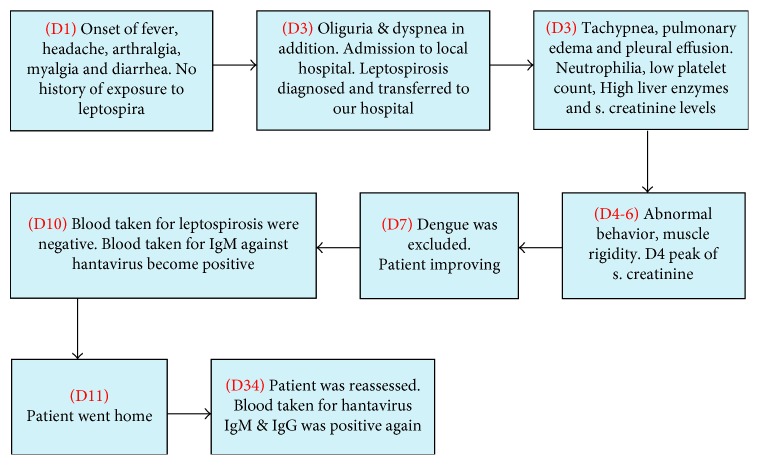
The timeline of salient events of case 2.

Figure 3 depicts the timeline of salient events of case 2.

**Figure 4:**
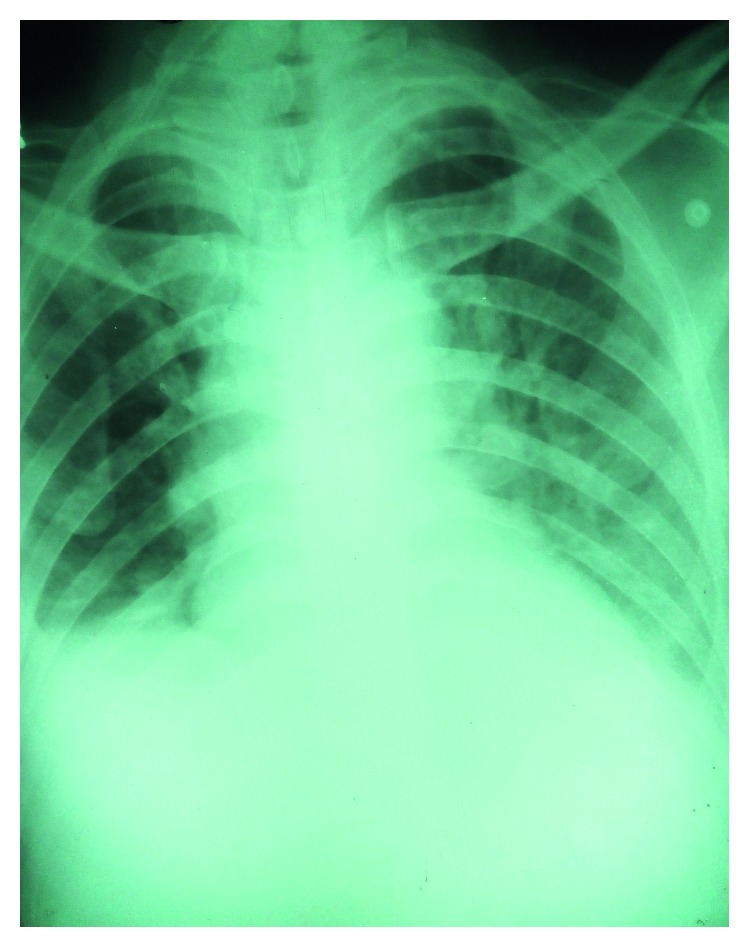
Chest X-ray of the patient of case 2 taken on day 4 of the illness showing patchy opacities more in the peripheries of both lung fields indicating pulmonary edema due to a noncardiac cause. A bilateral pleural effusion also can be seen.

A 57-year-old Sri Lankan Muslim (here, Muslim means his ethnic group) male presented to his local hospital in the Kegalle district on day 3 of the illness with fever, headache, arthralgia, and severe myalgia in legs. He had watery loose stools twice on day 2 and developed oliguria, cough, and dyspnea on day 3. He has been on treatment for epilepsy during the last 25 years and was on sodium valproate and topiramate. The doctors of that hospital have suspected leptospirosis and transferred the patient to our hospital for further management. There was no history suggestive of exposure to leptospira other than occasional sighting of a mouse at his shop. Physical examination upon admission showed a temperature of 39.5 degrees Celsius, mild dehydration, flushed skin, dyspnea, and bilateral mild ankle edema. His pulse rate was 110/minute; blood pressure was 160/100 mmHg; jugular venous pressure was elevated; respiratory rate was 24/minute, and there were bilateral fine crepitations in both lung fields; oxygen saturation was 90% while breathing room air. There were no other significant findings on system examinations. Diagnostic workup results showed a leukocyte count of 20.04 × 10^9^/l (91% neutrophils) and both leukocyte count and neutrophils percentage gradually declined. The platelet count was 116 × 10^9^/l and remained within 103–134 × 10^9^/l range during his hospital stay. There were no bleeding manifestations. CRP was 208.6 mg/dl, and blood and urine cultures (day 4) were negative. His ECG was normal and troponin I titre was 0.16 ng/ml (<0.12 ng/ml). His ALT level was 92.2 U/l and AST was 158.4 U/l. Total serum proteins were 5.21 g/dl (6.6–8.7 g/dl) and creatine kinase level was 240 mic/l (10–120 mic/l). NS1 antigen test for dengue (day 3) and IgM test for dengue on day 7 were negative. The day 4 chest X-ray (Figure 4) showed a bilateral small pleural effusion and pulmonary edema, and USS of the abdomen revealed edematous and thickened cortex of both kidneys and a bilateral pleural effusion.

Our working diagnosis was Weil's disease (leptospirosis). He was managed with supportive therapy (i.e., oxygen via nasal prongs, oral paracetamol) but not with mechanical ventilation and intravenous ceftriaxone. A 2D echocardiogram done on day 6 was normal with an ejection fraction of 60%. His day 3 serum creatinine level was 3.4 mg/dl and rose to 8.1 mg/dl on day 4 and then gradually reduced to 1.55 mg/dl on the day of his discharge (day 11). His oliguria and fluid overload responded well to intravenous furosemide and did not require any form of renal replacement therapy. An influenza A (H1N1) epidemic was going on at the time of his admission; hence, we covered that with oseltamivir. Oral clarythromycin was also administered to cover any scrub typhus infection because that is common in his locality. Throat and nasal swabs taken on day 5 were negative for influenza A (H1N1) viral RNA. Blood was taken for a microscopic agglutination test (MAT) for IgM and IgG against leptospira on day 10 and was also negative. Both these reports arrived after he was discharged. He had altered behavior between day 4 and 6 and had symmetrical generalized muscle rigidity and finger tremors on day 5 of the illness. On the night of day 4, he was given olanzapine and clonazepam orally as we will discuss below. Blood taken on day 10 was positive for IgM against hantavirus on 1 : 100 dilution, and blood taken on day 34 was positive for IgM against hantavirus on 1 : 100 and 1 : 500 dilutions and IFA for IgG against hantavirus as well.

## 3. Discussion

A hantavirus infection is very rarely diagnosed in Sri Lanka and in other South Asian countries, and many doctors are unfamiliar with it [4, 5]. Our case 1 may be the first reported case where a hantavirus infection was diagnosed whilst the patient was still being treated at a Sri Lankan hospital (in mid-2016) even though the first hantavirus infection of Sri Lanka was detected in 1998 [5, 6]. It is not possible to diagnose hantavirus infections, especially mild and moderate cases, without serology, especially in a setting where leptospirosis is endemic [1, 5]. We presume, because of this and due to the reasons given in the introduction, many hantavirus infections get misdiagnosed as leptospirosis in Sri Lanka and elsewhere like we also initially did [5]. There are two past reports from Sri Lanka where hantavirus infections were diagnosed retrospectively by detection of antibodies against hantaviruses in patients managed as leptospirosis [5, 6].

Both of our patients had renal involvement as well as lung involvement (a combination of clinical features of HFRS and HPS). In addition, CNS involvement was also there, especially as case 1 had encephalitis. There is extreme paucity of such case reports. However, an HPS and encephalitis combination has been reported in the past [7]. The EEG of case 1 was compatible with encephalitis; his CSF had high protein levels and lymphocytes indicating CNS infection, but high erythrocyte count as well indicating a traumatic tap. A high fever due to many infections also can give rise to abnormal behavior (delirium) [8]. Metronidazole and acyclovir are respectively known to cause encephalopathy and confusion rarely [9, 10]. Nonetheless, case 1 had CNS symptoms before starting these drugs; hence, we believe his infection caused the symptoms. On day 4, we sought psychiatric and neurological opinions regarding the abnormal behavior of case 2. The psychiatric team suggested olanzapine 5 mg and clonazepam 0.5 mg to be given nocte. The neurologist detected symmetrical muscle rigidity as well as cogwheel rigidity on day 5 and suspected olanzapine contributing to that. Hence, after the first dose, both drugs were omitted. The patient improved rapidly before we finalized the arrangement to perform an NCCT brain and an EEG. Hence, those were not done. The presence of abnormal behavior before the start of olanzapine indicates his infection affecting the brain as the likely original reason of his abnormal behavior. Nonetheless, we did not find solid evidence for encephalitis in case 2. A generalized increase of muscle tone with cogwheel rigidity has been reported in other viral infections as well [11]. Overlaps of clinical features between HPS and HFRS in other countries were described in recent review articles [3, 12]. The HPS case fatality rate in the Americas is as high as 40% [1, 2]. Despite coming to the correct diagnosis late/retrospectively, both these patients recovered notwithstanding being seriously ill (especially case 1). We saw their serology reports after the patients started to recover/after they went home. That indicates that an adequate supportive therapy can save the lives of such patients, yet a larger case series may be need for a conclusion.

New World hantaviruses that cause HPS are considered the highest risk (Biodefense Category A) pathogens within the antibioterrorism program of the National Institutes of Allergy and Infectious Diseases of the United States [2, 13]. Nevertheless, they consider dengue, the commonest infectious disease we see at most Sri Lankan hospitals, including ours at present, also as a Biodefense Category A pathogen. Therefore, there is no need to panic, but we shall be concerned because both these patients had clinical features of not only of HPS but also of HFRS plus CNS involvement as well. These unusual manifestations of the disease may be due to patient factors, virus factors, or a combination of both. The emergence of an infection seriously affecting multiple organ systems with high case fatality rate, spread by aerosol and other routes, is a serious public health issue. Nonetheless, hantavirus is not in the official notifiable diseases list of Sri Lanka [14]. We think that hantavirus infections deserve to be on that list. In a recent study done in Kandy, 2 out of 102 rats trapped were infected by a possibly new hantavirus strain [15]. Genetic reassortment of hantaviruses occurs in nature [3, 16]. With that background, it is possible that a hitherto unidentified hantavirus strain caused these two infections with atypical clinical features.

We believe it is worthwhile to consider hantavirus infection as a differential diagnosis in Sri Lankan patients, presenting with histories and clinical features suggestive of leptospirosis and in patients with a fever of unknown origin (FUO). As discussed above, serological tests or RT-PCR to detect hantavirus ribonucleic acid (RNA) are necessary for the diagnosis of the disease [1–3]. Making those tests available at major hospitals of the country will help clinicians to diagnose the disease and notify. Then, the public health teams can investigate the cases and control the spread of disease. Hantavirus produces chronic persistent infections in the host rodent, and it continues to spread the virus [1, 2]. Hence, giving more emphasis to rodent control, especially around dwellings of the detected/suspected cases, may help to control the spread of the disease. Sri Lanka is home to 23 rodent, 30 bat, and 10–11 shrew species [17]. Each hantavirus is specifically associated with a single species of mammal, which acts as its primary reservoir [1, 2]. At present, no information is available regarding any Sri Lankan bats, shrews (soricomorphs), and most rodent species' roles in hosting hantavirus. Seroprevalence studies for hantavirus among those mammals would also be useful to clearly understand the epidemiology of the infection. A vaccine against hantavirus is available and the antiviral drug ribavirin (Ribavin) is used in countries like China but is unavailable locally [1, 2]. Ribavirin is in the World Health Organization's (WHO) current (2017) model list of essential medicines, and it has been in the list for more than 10 years [1, 18]. Making ribavirin available in Sri Lanka may be useful in treating seriously ill patients with HFRS. However, ribavirin is ineffective in treating HPS [1, 2]. As we have described above and as mentioned in one review article, adequate supportive therapy is very important in managing such patients [3]. Existing vaccines against hantavirus are not WHO approved. We have administered methylprednisolone 1 mg intravenously daily from day 5–7 to the case 1 presuming pulmonary involvement of leptospirosis as per our current national guidelines [19, 20]. However, we did not prescribe that for case 2 as we suspected influenza A (H1N1) as one of the differential diagnosis [21]. Corticosteroids are used to treat severe hantavirus infections [1, 2]. Methylprednisolone may have contributed to his recovery. However, a double-blind, randomized controlled clinical trial has demonstrated that methylprednisolone did not provide significant clinical benefit to patients with Andes virus-related HPS in Chile [22].
